# Palliative Sedation—The Last Resort in Case of Difficult Symptom Control: A Narrative Review and Experiences from Palliative Care in Switzerland

**DOI:** 10.3390/life12020298

**Published:** 2022-02-16

**Authors:** Cristian Camartin, Linda Björkhem-Bergman

**Affiliations:** 1 Palliative Care, Kantonsspital Graubünden, Loestrasse 170, CH-7000 Chur, Switzerland; 2 Division of Geriatrics, Department of Neurobiology, Care Sciences and Society, Karolinska Institutet, Blickagången 16, SE-141 83 Huddinge, Sweden; linda.bjorkhem-bergman@ki.se; 3 Palliative Care, Stockholms Sjukhem, Mariebergsgatan 22, SE-112 19 Stockholm, Sweden

**Keywords:** palliative sedation, ethical issues, symptom management, euthanasia, palliative care, midazolam

## Abstract

Palliative sedation can be considered as “the last resort” in order to treat unbearable, refractory symptoms or suffering in end-of-life patients. The aim is symptom relief and not to induce death as in the case of euthanasia. The treatment might be one of the most challenging therapeutic options in the field of palliative care, involving both ethical and practical issues. Still, studies have shown that it is a safe and valuable treatment and in general does not shorten the life of the patient. Since patients in Switzerland have the legal option of assisted suicide, palliative sedation is an alternative that has become increasingly important. The use of palliative sedation was reported in 17.5% of all patients admitted to palliative care in Switzerland, making the country of those with the highest use of this treatment. The aim of this narrative review is to discuss ethical and practical issues in palliative sedation, with specific focus on experiences from Switzerland. Indications, ethical considerations, drugs of choice and duration are discussed. Decision making should be based on solid guidelines. When used correctly, palliative sedation is an important and useful tool in palliative care in order to provide good symptom relief.

## 1. Introduction

Palliative sedation may be the last resort in case of difficult symptom control at the end of life. In 2009, the European Association for Palliative Care (EAPC) defined palliative sedation as “the monitored use of medications intended to induce a state of decreased or absent awareness (unconsciousness) to relieve the burden of otherwise intractable suffering in a manner that is ethically acceptable to the patient, family and health-care providers” [1]. Thus, the aim of palliative sedation is symptom relief and the intention is not to shorten life or induce death, in contrast to euthanasia. It is a treatment that belongs to the basic skills of a palliative care physician and should also be applied when necessary [2].

The aim of this narrative review is to discuss ethical and practical issues in palliative sedation, with specific focus on experiences from Switzerland. This review is based on our own experiences from palliative care and does not claim to be fully comprehensive. We do not present a systematic literature review but rather a selection of key references that we think highlight important aspects in this topic.

Since patients in Switzerland have the legal option of assisted suicide, palliative sedation is an alternative that has become increasingly important. In the case of progressive disease, symptoms can lead to the impairment of abilities and thus result in a lack of autonomy for the individual. If these symptoms cannot be adequately treated at the end of life, the use of palliative sedation can be considered. However, according to our own experiences, palliative sedation is one of the most challenging therapeutic options in the field of palliative care.

The topic of palliative sedation was discussed emotionally in Swiss media a few years ago [3]. It was clear that different terms—euthanasia, assisted suicide and palliative sedation—were mixed up and everything was described as “euthanasia”. The clear distinction between palliative sedation and euthanasia is important to emphasize and is further discussed and explained in Section 4.2.1 below.

Palliative sedation affects the whole spectrum of palliative care, namely the somatic, psychological, social, spiritual, and cultural aspects. Thus, according to our own experiences, palliative sedation is very demanding for the treatment team. Thus, the use of solid guidelines in the process of decision making, documentation and evaluation is very valuable.

## 2. Method

The literature search for this review was performed in PubMed and based on different combinations of the text words and MeSH terms “palliative sedation”, “euthanasia”, “conscious sedation/methods”, “deep sedation/methods”, “palliative care/methods”, “terminal care/methods”, “symptom management/method” and “guideline adherence”. In addition, we added articles from national journals in Switzerland and Sweden.

We evaluated the text of this review by using the Scale for the Assessment of Narrative Review Articles (SANRA) score [4].

## 3. Frequency of Palliative Sedation

The frequency of palliative sedation, defined as the number of patients receiving this treatment of all patients enrolled in palliative care, differs markedly around the world. The definition of palliative sedation might differ between studies and thus the figures presented in different studies might not be totally comparable. In a Swedish study from 2009, less than 1% admitted to a palliative care unit received palliative sedation [5]. The same low number, 1%, was reported in Japan [6]. In contrast, the frequency was 15% in a study performed in the USA [7], 17.5% in Switzerland [8] and 18% in the Netherlands [9].

In recent years, the use of palliative sedation seems to be increasing [8,10]. In the Netherlands, there was an increase from 8.2% of all deaths in 2005 to 18.3% in 2015 [10]. In Switzerland, there was an increase from 4.7% in 2001 to 17.5% in 2013, i.e., a 4-fold increase in 12 years [8]. Likewise, in a recent study performed in Sweden, the use of palliative sedation was much higher than was reported 10 years ago (Hedman C and co-workers, unpublished data [5]).

In Switzerland, four different languages and cultures can be distinguished—German, French, Italian and Romansh—all characterized by their own customs and practices. According to a study from 2018, the use of palliative sedation differs among these cultures [11]. Palliative sedation was used more frequently in the Italian-speaking region compared to the French- and German-speaking regions. Interestingly, the frequency of assisted suicide, which might be considered as an alternative to palliative sedation, was similar in the German- and French-speaking regions (1.6% and 1.2%) but no cases were reported in the Italian-speaking region. An increase in the number of palliative sedations can certainly be attributed to the growing awareness of palliative care, the more intensive treatment of dying patients, and possibly also the increasing awareness and perception of this treatment option [10].

## 4. Ethical Issues

The Swiss context might be particularly interesting regarding end-of-life policies. Assisted suicide is practiced in this country because of a law stating that assisting suicide for non-egoistic motives is not punishable. The law does not explicitly mention physician-assisted suicide, whereas euthanasia is strictly prohibited [12]. This is in contrast to the situation in, e.g., the Netherlands, Belgium, Luxembourg, Western Australia, Canada, and Spain, where euthanasia is fully legalized [13]. According to ethical principles, a patient’s therapy should be chosen in such a way that the four ethical principles according to Beauchamp and Childress are observed. These are described as follows: respect for autonomy; non-maleficence; beneficence; and justice [12]. In the case of palliative sedation, all four principles must be observed.

### 4.1. Respect for Autonomy

According to EAPC guidelines, the decision to start palliative sedation should be made in accordance with the patient’s wishes; or, when this is not possible, in consultation with a surrogate decision maker [1]. It is well known that patients in Switzerland want to have a say in treatment and be involved in decision-making processes [14]. The option of palliative sedation should therefore be discussed with patients as early as possible, ideally before acute crises, and this option should be explained to them. The patient or their representative must give their consent for this to be carried out. It has been shown that palliative sedation is rarely an emergency treatment but is a targeted treatment in the case of untreatable, refractory symptoms.

### 4.2. Non-Maleficence

Occasionally, palliative sedation leads to the question of whether the patient will be harmed by shortening his or her life. Notably, all studies performed on this topic have shown that life was never assessed to be shortened by palliative sedation [15,16]. However, there are of course some methodological limitations in such studies since randomized, controlled studies can never be performed due to ethical reasons. Interestingly, in contrast to the fear that an acceleration of the dying process could occur, a prolongation of the life span has been reported [17]. The reason for this seems to be a reduction in stress experienced by the patient [18]. In this context and in order to avoid harm, distinguishing and defining palliative sedation are of central importance. Euthanasia, which is legally possible in certain countries, must be clearly distinguished from palliative sedation. The aim of euthanasia is to end the patient’s life as quickly as possible. Palliative sedation, on the other hand, aims to alleviate the patient’s suffering and in no way seeks to end life. Of course patients meeting criteria for palliative sedation will also die, but this will be as a consequence of the underlying disease and not due to palliative sedation. For patients who express a wish for assisted suicide, it is discussed whether palliative sedation should be offered as an alternative. We find that, on the contrary, palliative sedation should be an independent treatment with a focus on relieving symptoms [19].

#### 4.2.1. Palliative Sedation versus Euthanasia

The EAPC distinguishes between palliative sedation and euthanasia in terms of intention, procedure, and result [20]. The intention of the care in palliative sedation is to provide relief from unbearable suffering and/or refractory symptoms; in euthanasia, the intention is death of the patient. The procedure in palliative sedation involves the individualized, monitored and titrated use of sedative drugs to control refractory symptoms; in euthanasia, the same fixed and standard doses of lethal drugs are used for all patients, without individualization. In palliative sedation, the “successful” result of the treatment is symptom relief; in euthanasia, the outcome is “successful” when the patient dies.

### 4.3. Beneficence

As with all medical care, the patient’s well-being should be in the foreground, and this should also be the indication for palliative sedation. Criteria for palliative sedation in Switzerland can be manifold and essentially do not differ from those of other countries—the focus is on avoiding delirium at the end of life, reducing respiratory distress, and ameliorating pain [21,22]. With treatment initiated in this way, these symptoms can be well controlled.

### 4.4. Justice

In principle, palliative sedation is not reserved for palliative care units but can also be offered on internal medicine wards or even at home with the help of an outpatient palliative care service. The treatment should be offered to all patients who can benefit from it and should not only be used in specialized centers [23].

According to a Cochrane analysis involving 4167 patients, of whom 1137 received palliative sedation, 95% of patients treated with palliative sedation were suffering from cancer [15]. This indicates that patients with non-oncological diseases may not have had the same opportunity to obtain palliative sedation.

The final decision to begin palliative sedation is made by a senior physician, preferably a palliative care physician, but should always be discussed with other health care professionals within the team. The physician is also responsible for prescribing drugs, communicating with the patient and family and documentation in medical records. However, the whole care team is involved in this process and supports the patient in different ways.

## 5. Practical Issues—Indication, Medication and Duration

### 5.1. Indications

Palliative sedation should be considered in the case of unbearable suffering. The assessment and evaluation of whether suffering is unbearable should be made primarily by the patient or, in the case of loss of capacity, by a representative [24]. Suffering can exist in a wide variety of areas—somatic, psychological, social, or even spiritual suffering can be prevalent. Whether suffering is considered unbearable for the individual patient is a purely subjective assessment and can therefore lead to controversy among the treatment team. As assisted suicide is an option in Switzerland and, unlike in the Netherlands, Belgium, and Luxembourg, euthanasia is prohibited, palliative sedation may be considered in the case of exceptional, refractory discomfort. EAPC guidelines document the following indications for the use of palliative sedation: (1) transient sedation for noxious procedures, (2) sedation as part of burn care, (3) sedation used for end-of-life weaning from ventilator support, (4) sedation in the management of refractory symptoms at the end of life, (5) emergency sedation, (6) respite sedation and (7) sedation for psychological or existential suffering.

In recent years, an increase in palliative sedation has been observed [8,10]. One possible explanation for this is that the indication for use has been expanded from somatic to non-somatic complaints. In recent years, use has been expanded to include symptoms such as fear, anxiety and existential stress [9]. This approach is agreed to by a majority of physicians [25]. Among somatic complaints, mainly three symptoms are considered for palliative sedation—delirium, breathlessness, and pain [26]. Patients often fear the end of life and the dying process. The symptoms that are mainly seen as indications, such as breathlessness, and pain, are also the symptoms that seem most threatening to patients and trigger anxiety [27].

### 5.2. The Value of Guidelines

The practical application of palliative sedation should be based on the guidelines. Various versions are available, but they are substantially similar. A very common recommendation is that of the European Association for Palliative Care (EAPC) [1]. These guidelines emphasize that the decision making to start palliative sedation should be based on input from the multi-professional palliative care team, rather than by the treating physician alone. In addition, the medical rationale for recommending sedation, the decision-making process, the aims of sedation, the planned depth and duration of sedation should be thoroughly recorded in the patient’s medical record in order to be easily accessible for the health care professionals taking care of the patient. The decision to start palliative sedation and the aims, benefits and risks of the proposed sedation should be discussed with the patient, if possible, and with the next of kin. In several cases, the patients lack decisional capacity and the decision has to be discussed with their next of kin only. It should be stressed that the role of the next of kin is not to decide, but rather to indicate what the patient would have wanted and why. It is important that the next of kin is assured that the professional care team takes the responsibility for the medical decision [1].

In contrast to most other countries, there is only one published guideline for palliative sedation in Switzerland [28]. This was published by the Swiss Society for Palliative Medicine, the so-called Bigorio guideline, named after the place of origin and compiled by a group of experts based on international guidelines [29].

### 5.3. Drugs

The most commonly used drug used for palliative sedation around the world is continuously administrated midazolam [5,8,9,15,16]. It has a rapid onset of action and is considered as safer and more tolerable than most other sedatives. Due to its short half-life, administration by continuous infusion, subcutaneously or intravenously, is generally required to maintain a sustained effect. However, the brief duration is also an advantage, making it is easier to adjust to a sufficient dose. Bolus doses can be given in crisis situations.

Other drugs used for palliative sedation include haloperidol and other neuroleptics, benzodiazepines (in addition to midazolam) and propofol [9,15,16]. Propofol is mostly used when midazolam is insufficient [30]. Opioids have also been used for palliative sedation in different settings [16,31,32]. However, the use of opioids for sedation has been questioned since sedation is a secondary effect of opioids, rather than the primary effect [16].

According to EAPC guidelines, the level of sedation should be the lowest dose required to provide sufficient symptom relief [1]. However, the guidelines also state that deeper sedation may be necessary when (1) suffering is intense, (2) suffering is refractory, (3) death is anticipated within hours or a few days, (4) the patient’s wish is explicit or (5), in the setting of an end-of-life catastrophic event, such as massive hemorrhage or asphyxia.

In Switzerland, in concordance with other countries, midazolam is the most commonly used drug for sedation. In rarer cases or as second-line medication, neuroleptics such as levomepromazine, or hypnotics, such as propofol, are used. No opioid has been used as the sole sedative medication in any specialized palliative care center, in contrast to non-specialized palliative care [26]. Considering the depth of knowledge available on the indications and effects of opioids, it is likely that they were mainly used as medication for existing complaints, such as pain and breathlessness, but not directly as sedatives. This again shows the need to define palliative sedation. In specialized palliative care, palliative sedation is defined as continuous deep sedation; in primary care, occasionally even the single, sole administration of a peroral benzodiazepine is regarded as sedation.

### 5.4. Duration of Sedation

An important question that always arises is when palliative sedation should be initiated. Basically, after evaluation within the treatment team and in consultation with the patient and his relatives, a precise indication for its use should be discussed. In view of the underlying disease, various symptoms may be expected. If these pre-determined symptoms occur, palliative sedation is justified and should be initiated. The duration of sedation varies significantly from individual to individual and can range from a few minutes to several days. Various studies could show that the fear of a shortening of life with the use of palliative sedation is not justified [15,16,17]. On the contrary, a tendency towards longer life was shown [17]. The reason for this is probably a reduction in stress experienced by the patient [17]. A stressful situation with distressing symptoms can lead to a strain on the entire body and thus be a detriment to the entire organism. Sedation should be given at the necessary level so that patients have good symptom control. As the disease progresses, dose reduction should be considered but not routinely attempted [33]. The duration of sedation cannot be determined in advance.

In a systematic review including ten studies, the mean duration of palliative sedation ranged from 0.8 to 12.6 days [16]. In contrast, in a review including six studies on palliative sedation at home, the duration ranged from 1 to 3.5 days [34].

In specialized palliative care in Switzerland, the duration of palliative sedation is reported to be approximately 1 to 2 days [21]. Sedation is managed based on discomfort. If justified, sedation can be continued until the patient dies [35].

## 6. Discussion

The challenges in palliative sedation have been described in other cultural contexts before, e.g., reports from the USA, Mexico, Belgium, the Netherlands, Japan, Singapore, Germany and the United Kingdom [25,36,37]. Difficulty in communicating the differences between palliative sedation and physician-assisted suicide or euthanasia seems to be a problem in most countries. The fear of shortening the patient´s life is also a concern raised in most reports. Generally, the use of palliative sedation seems to be more accepted for management of refractory physical symptoms than for psycho-existential suffering [36].

In Switzerland, the use of palliative sedation is a regularly performed treatment in palliative care. In specialized palliative care, a temporary or permanent reduction in consciousness with the help of sedative medication to treat a wide variety of stressful situations is referred to as palliative sedation. It is important to define palliative sedation correctly and to clarify the differences between palliative sedation euthanasia or assisted suicide. This intervention option represents a very demanding treatment for the treatment team but also for the patient and their relatives. The entire spectrum of palliative care is involved, i.e., somatic, psychological, social, spiritual, and cultural aspects need to be taken into account.

It has been shown that palliative sedation is very safe if guidelines and directives are followed [15,17]. In particular, the fear that it could lead to a shortening of life and thus to an acceleration of the dying process, or that it is euthanasia, could be refuted. Even small doses of a sedative, mostly midazolam, could show the desired success. The duration of sedation should be as short as possible but adequately long. When palliative sedation is started, it is important to continue the symptomatic treatment already initiated in order to not risk an increase in symptoms. However, medication should be limited to substances that are indicated in order to prevent unnecessary medication. All treatments and corresponding procedures should be discussed in detail with the patient and their relatives to avoid misunderstandings. Discontinuation of the administration of additional fluids and nutrition should be well communicated, as these treatments are of secondary importance in terminal sedation and should be terminated.

## 7. Conclusions

In conclusion, the use of palliative sedation, including indication, ethical considerations, choice of medication and duration, should be based on guidelines and thoroughly followed up and evaluated regularly. When used correctly, palliative sedation is an important and valuable tool in palliative care in order to provide good symptom control, which is the goal in palliative care.

**Figure 1:**
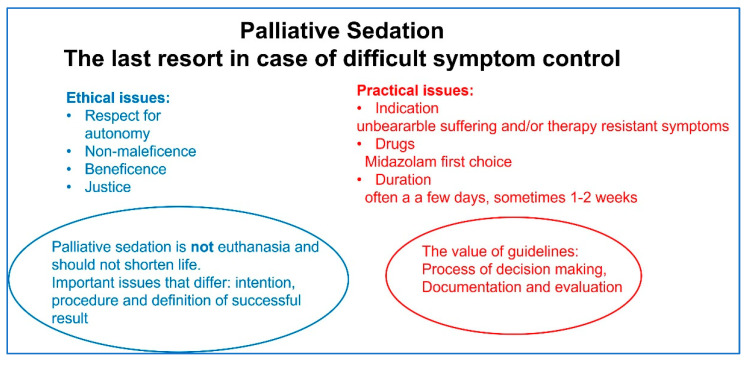
Summary of ethical and practical issues in palliative sedation.

Key notes from this narrative review are summarized below (Figure 1).

## Data Availability

Not applicable.
